# Selecting subsets of newly extracted features from PCA and PLS in microarray data analysis

**DOI:** 10.1186/1471-2164-9-S2-S24

**Published:** 2008-09-16

**Authors:** Guo-Zheng Li, Hua-Long Bu, Mary Qu Yang, Xue-Qiang Zeng, Jack Y Yang

**Affiliations:** 1 Department of Control Science & Engineering, Tongji University, Shanghai 201804, PR China; 2 School of Computer Science & Engineering, Shanghai University, Shanghai 200072, PR China; 3 National Human Genome Research Institute National Institutes of Health (NIH) U.S., Department of Health and Human Services Bethesda, MD 20852, USA; 4 Harvard Medical School, Harvard University, Cambridge, MA 02140-0888, USA

## Abstract

**Background:**

Dimension reduction is a critical issue in the analysis of microarray data, because the high dimensionality of gene expression microarray data set hurts generalization performance of classifiers. It consists of two types of methods, i.e. feature selection and feature extraction. Principle component analysis (PCA) and partial least squares (PLS) are two frequently used feature extraction methods, and in the previous works, the top several components of PCA or PLS are selected for modeling according to the descending order of eigenvalues. While in this paper, we prove that not all the top features are useful, but features should be selected from all the components by feature selection methods.

**Results:**

We demonstrate a framework for selecting feature subsets from all the newly extracted components, leading to reduced classification error rates on the gene expression microarray data. Here we have considered both an unsupervised method PCA and a supervised method PLS for extracting new components, genetic algorithms for feature selection, and support vector machines and *k *nearest neighbor for classification. Experimental results illustrate that our proposed framework is effective to select feature subsets and to reduce classification error rates.

**Conclusion:**

Not only the top features newly extracted by PCA or PLS are important, therefore, feature selection should be performed to select subsets from new features to improve generalization performance of classifiers.

## Background

Tumor classification is performed on microarray data collected by DNA microarray experiments from tissue and cell samples [1-3]. The wealth of this kind of data in different stages of cell cycles helps to explore gene interactions and to discover gene functions. Moreover, obtaining genome-wide expression data from tumor tissues gives insight into the gene expression variation of various tumor types, thus providing clues for tumor classification of individual samples. The output of microarray experiment is summarized as an *n *× *p *data matrix, where *n *is the number of tissue or cell samples; *p *is the number of genes. Here *p *is always much larger than *n*, which hurts generalization performance of most classification methods. To overcome this problem, dimension reduction methods are applied to reduce the dimensionality from *p *to *q *with *q *≪ *p*.

Dimension reduction usually consists of two types of methods, feature selection and feature extraction [4]. Feature selection chooses a subset from original features according to classification performance, the optimal subset should contain relevant but non redundant features. Feature selection can help to improve generalization performance and speed of classifiers. There have been a great deal of work in machine learning and related areas to address this issue [5-9]. But in most practical cases, relevant features are not known beforehand. Finding out which features to be used is a hard work. At the same time, feature selection will lose the relevant information among features, while feature extraction is good at handling interactions among features.

Feature extraction projects the whole data into a low dimensional space and constructs the new dimensions (components) by analyzing the statistical relationship hidden in the data set. Principle components analysis (PCA) is one of the frequently used methods for feature extraction of microarray data. It is unsupervised, since it need not the label information of the data sets. Partial Least Squares (PLS) is one of the widely used supervised feature extraction methods for analysis of gene expression microarray data [10,11], it represents the data in a low dimensional space through linear transformation. Although feature extraction methods produce independent features, but Usually, a large number of features are extracted to represent the original data. As we known, the extracted features also contain noise or irrelevant information. Choosing an appropriate set of features is critical. Some researcher considered that the initial several components of PLS contain more information than the others, but it is hard to decide how many tail components are trivial for discrimination. Some authors proposed to fixed the number of components from three to five [12]; some proposed to determine the size of the space by classification performance of cross-validation [13]. However each one has its own weakness. Fixing at an arbitrary dimensional size is not applicable to all data sets, and the cross-validation method is often obstructed by its high computation. An efficient and effective model selection method for PLS is demanded. Furthermore, we consider not all the initial components are important for classification, subsets should be selected for classification.

Here, we propose and demonstrate the importance of feature selection after feature extraction in the tumor classification problems. We have performed experiments by using PCA [14] and PLS [15] as feature extraction methods separately. In this paper, we will perform a systematic study on both PCA and PLS methods, which will be combined with the feature selection methods (Genetic Algorithm) to get more robust and efficient dimensional space, and then the constructed data from the original data is used with Support Vector Machine (SVM) and *k *Nearest Neighbor (*k*NN) for classification. By applying the systematic study on the analysis of gene microarray data, we try to study whether feature selection selects proper components for PCA and PLS dimension reduction and whether only the top components are nontrivial for classification.

## Results and discussion

### Results by using SVM

In order to demonstrate the importance of feature selection in dimension reduction, we have performed the following series experiments by using support vector machine (SVM) as the classifier:

1. SVM is a baseline method, all the genes without any selection and extraction are input into SVM for classification.

2. PCASVM uses PCA as feature extraction methods, all the newly extracted components are input into SVM.

3. PLSSVM uses PLS as feature extraction methods, all the newly extracted components are input into SVM.

4. PPSVM uses PCA+PLS as feature extraction methods, all the newly extracted components are input into SVM.

5. GAPCASVM uses PCA as feature extraction methods to extract new components from original gene set and GA as feature selection methods to select feature subset from the newly extracted components, the selected subset is input into SVM.

6. GAPLSSVM uses PLS as feature extraction methods to extract new components from original gene set and GA as feature selection methods to select feature subset from the newly extracted components, the selected subset is input into SVM.

7. GAPPSVM uses PCA+PLS as feature extraction methods to extract new components from original gene set and GA as feature selection methods to select feature subset from the newly extracted components, the selected subset is input into SVM.

Since there are parameters for SVM, we try to reduce its effect to our comparison and use four pairs of different parameters for SVM, they are *C *= 10, *σ *= 0.01, *C *= 10, *σ *= 10, *C *= 1000, *σ *= 0.01, and *C *= 1000, *σ *= 10. It is noted that different data sets including the extracted data sets and selected data sets need different optimal parameters for different methods, we do not choose the optimal parameters, because 1) this is unreachable, finding the optimal parameters is an NP hard problem; 2) we do not exhibit the top performance of one special method on one single data set, but we want to show the effect of our proposed framework.

#### Prediction performance

The average error rates and the corresponding standard deviation values are shown in Table 1, where the standard deviation values are produced from our 50 times repeated experiments. From Table 1, we can find that:

**Table 1:** Statistical classification error rates (and their corresponding standard deviation) by using SVM with different parameters on four microarray data sets (%)

DATASET	SVM	PCASVM	GAPCASVM	PLSSVM	GAPLSSVM	PPSVM	GAPPSVM
*C *= 10, *σ *= 0.01 for SVM							

CNS	40.4(8.6)	36.4(4.4)	35.2(1.6)	36.6(2.8)	35.0(5.6)	36.2(5.3)	35.0(7.0)
COLON	34.6(5.4)	31.3(7.0)	29.6(7.5)	29.5(8.2)	28.8(9.2)	33.2(6.7)	30.5(7.2)
LEUKEMIA	27.0(7.1)	26.2(6.1)	23.2(6.4)	20.4(7.0)	16.2(5.9)	22.9(7.5)	21.7(7.2)
LUNG	4.2(7.2)	4.1(5.9)	3.9(6.2)	3.7(6.2)	3.3(5.8)	4.4(5.5)	4.0(7.3)
Average	26.8(7.1)	24.5(5.9)	22.9(5.4)	22.5(6.1)	20.8(6.7)	24.1(6.2)	22.8(7.2)

*C *= 10, *σ *= 10 for SVM							

CNS	40.6(9.2)	35.7(4.2)	34.4(7.3)	39.6(7.5)	38.4(5.5)	40.0(5.3)	38.7(8.3)
COLON	34.6(5.4)	29.1(8.3)	29.6(7.3)	29.7(8.3)	29.1(9.3)	33.7(6.9)	32.2(7.2)
LEUKEMIA	27.4(6.9)	26.3(6.2)	22.1(6.3)	20.4(7.0)	16.3(6.0)	22.8(7.3)	20.9(7.2)
LUNG	3.7(6.9)	2.9(6.2)	1.7(6.2)	1.6(6.8)	1.6(6.1)	1.6(8.5)	1.5(4.4)
Average	26.5(7.1)	23.4(6.2)	21.9(6.8)	22.8(7.4)	21.3(6.7)	24.5(6.9)	23.3(6.8)

*C *= 1000, *σ *= 0.01 for SVM							

CNS	37.6(7.6)	35.4(1.7)	34.4(1.0)	36.1(2.9)	35.0(5.4)	36.0(5.0)	35.6(3.1)
COLON	33.7(5.2)	31.0(7.8)	29.9(7.3)	28.9(8.7)	27.8(9.3)	33.4(7.2)	30.1(8.0)
LEUKEMIA	26.8(7.2)	25.8(6.2)	23.3(6.3)	20.5(6.9)	16.4(9.5)	24.0(7.4)	21.0(7.1)
LUNG	4.2(6.9)	4.1(6.5)	3.2(6.2)	3.4(7.2)	3.4(8.4)	4.0(6.3)	3.3(5.8)
Average	25.6(6.8)	24.0(5.5)	22.7(5.2)	22.2(6.4)	20.6(8.2)	24.6(6.5)	22.9(6.0)

*C *= 1000, *σ *= 10 for SVM							

CNS	41.4(8.7)	35.4(8.6)	34.0(8.4)	40.9(7.6)	38.9(5.8)	42.5(9.1)	39.2(8.8)
COLON	33.9(6.0)	31.0(7.5)	29.7(7.0)	29.0(8.7)	28.5(9.3)	32.8(7.2)	29.7(8.0)
LEUKEMIA	27.9(7.2)	25.0(6.1)	23.1(6.6)	20.5(6.9)	16.4(5.9)	22.6(7.7)	21.1(7.3)
LUNG	3.9(5.8)	2.8(6.5)	1.3(6.8)	1.7(6.3)	1.4(6.9)	3.6(6.3)	1.3(6.4)
Average	26.7(6.9)	23.5(7.2)	22.0(7.2)	23.0(7.3)	21.2(7.0)	25.4(7.6)	22.8(7.6)

• Results of all the classification methods with feature selection and extraction like PLSSVM, GAPLSSVM, PCASVM, GAPCASVM, GAPPSVM are better than that of SVM without any dimension reduction on average. Only on the LUNG data set, when SVM uses parameters of *C *= 10, *σ *= 0.01, results of PPSVM are worse than those of SVM.

• Results of classification methods with feature selection like GAPLSSVM, GAPCASVM and GAPPSVM are better than those of the corresponding feature extraction methods without feature selection like PLSSVM, PCASVM and PPSVM on average. Only on few cases, i.e. when *C *= 10, *σ *= 10 is for SVM, results of GAPCASVM are slightly worse than those of PCASVM on the COLON data set.

• Results of GAPLSSVM are better than those of PCASVM and GAPCASVM, even the corresponding results of PPSVM and GAPPSVM on average. Only on the CNS data set out of four data sets, GAPCASVM obtains the best results than other methods do.

• Results of PPSVM and GAPPSVM which combine PCA and PLS as feature extraction methods are not the best, just equal with those of PCASVM and GAPCASVM.

#### Number of selected features

We also show the number of features selected by each method with their corresponding standard deviation in Table 2, where the standard deviation values are produced from our 50 times repeated experiments. The values for PCASVM means the ratios of the number of top principle components to that of extracted components, those of PLSSVM and PPSVM have the same meaning. The values for GAPCASVM means the ratios of the number of selected components used in SVM to that of extracted components, and those of GAPLSSVM and GAPPSVM have the same meaning. From Table 2, we can see that if we use the top components, about 60–80% components are selected into learning machines, while if we use feature selection to select useful components, about 30% components are selected on average. Only on the LUNG data set, the selected components by different methods are 70%–80% of extracted components.

**Table 2:** Average percentage of features (and their corresponding standard deviation) used by SVM with different parameters on four microarray data sets (%)

DATASET	PCASVM	GAPCASVM	PLSSVM	GAPLSSVM	PPSVM	GAPPSVM
*C *= 10, *σ *= 0.01 for SVM						

CNS	74.4(8.5)	27.3(8.3)	67.7(10.0)	29.4(8.4)	68.8(6.6)	30.8(8.6)
COLON	81.1(7.5)	28.5(8.9)	57.6(4.8)	30.8(7.4)	59.4(9.2)	31.1(7.4)
LEUKEMIA	78.0(9.8)	26.7(6.3)	46.8(10.1)	30.0(6.5)	52.0(9.3)	30.3(6.6)
LUNG	82.2(6.2)	74.7(6.2)	73.8(7.9)	72.0(3.6)	82.3(7.6)	73.3(5.7)
Average	78.9(8.0)	39.3(7.4)	61.4(8.2)	40.5(6.5)	65.6(8.2)	41.3(7.1)

*C *= 10, *σ *= 10 for SVM						

CNS	73.4(9.8)	27.4(8.0)	62.7(9.0)	29.6(8.5)	65.7(9.3)	20.7(8.3)
COLON	82.1(6.5)	28.7(8.9)	57.6(4.8)	30.6(7.3)	59.3(8.3)	31.1(7.5)
LEUKEMIA	87.0(9.1)	26.7(6.1)	46.8(10.0)	30.0(6.3)	49.3(8.3)	30.4(6.7)
LUNG	77.4(7.0)	74.4(6.7)	76.3(6.9)	73.1(3.0)	83.1(8.1)	72.0(6.3)
Average	79.4(8.1)	39.3(7.4)	60.8(7.7)	40.8(6.3)	64.4(8.5)	38.5(7.2)

*C *= 1000, *σ *= 0.01 for SVM						

CNS	78.1(8.0)	27.4(8.2)	64.3(9.0)	29.3(8.3)	70.1(9.1)	30.6(8.3)
COLON	80.9(7.4)	28.1(8.6)	57.6(5.2)	30.7(7.3)	62.2(8.2)	31.0(7.5)
LEUKEMIA	87.1(9.7)	27.0(6.7)	47.6(10.0)	30.4(7.3)	49.2(8.3)	30.4(6.8)
LUNG	79.0(6.6)	76.8(6.3)	77.2(7.1)	67.4(4.3)	84.4(7.9)	81.4(6.6)
Average	81.3(7.9)	39.8(7.4)	61.6(7.8)	39.4(6.8)	66.4(8.4)	43.3(7.3)

*C *= 1000, *σ *= 10 for SVM						

CNS	76.2(8.9)	27.4(8.4)	67.1(8.9)	29.2(7.7)	69.4(9.1)	30.6(8.3)
COLON	82.5(7.2)	28.0(8.7)	59.5(4.6)	30.7(7.3)	63.3(8.7)	32.2(7.4)
LEUKEMIA	88.2(9.7)	27.3(6.7)	47.6(8.1)	30.3(7.3)	49.2(7.9)	30.4(6.8)
LUNG	81.1(6.8)	78.2(6.1)	81.3(5.1)	77.6(8.2)	81.1(8.0)	76.4(5.9)
Average	82.0(8.1)	40.2(7.0)	63.8(6.7)	41.9(7.6)	65.7(8.4)	42.4(7.1)

#### Distribution of selected features

**Figure 1:**
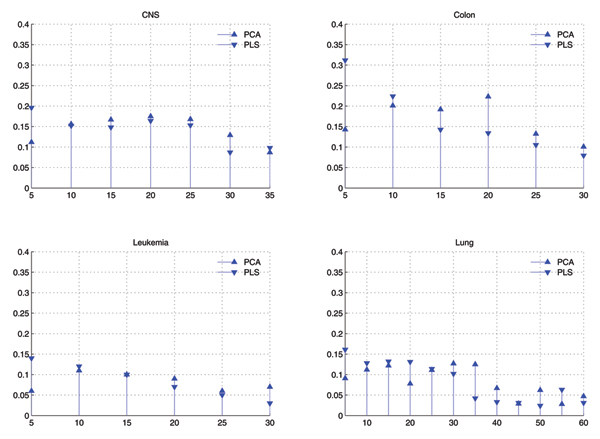
**Comparison of distributions of eigenvectors used by GAPCASVM and GAPLSSVM with *C *= 10, *σ *= 0.01 for SVM**. X-axis corresponds to the eigenvectors in descending order by their eigenvalues and has been divided into bins of size 5. Y-axis corresponds to the average value of times that eigenvectors within some bin are selected by GA.

**Figure 2:**
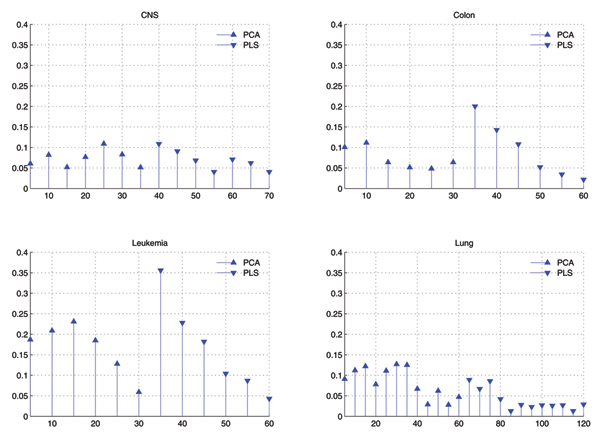
C**omparison of distributions of eigenvectors used by GAPPSVM with *C *= 10, *σ *= 0.01 for SVM**. X-axis corresponds to the eigenvectors in descending order by their eigenvalues and has been divided into bins of size 5. Y-axis corresponds to the average value of times that eigenvectors within some bin are selected by GA.

Fig. 1 shows the comparison of distributions of components selected by GA in two cases of GAPCASVM and GAPLSSVM, and Fig. 2 shows that of GAPPSVM. Difference between Fig. 1 and Fig. 2 is that in Fig. 1, PCA and PLS are used as feature extraction individually, while in Fig. 2, PCA is combined with PLS as feature extraction methods.

From Fig. 1 and Fig. 2, we can find that:

• When only PLS is used for feature extraction, the top components are a little more than that of others in the selected components, but the others are also important.

• When only PCA is used, the top components is less than others in the selected features, and the tail components are more important than others.

• When both PCA and PLS are used as feature extraction methods, they are nearly equal in the selected components, and the top components of PLS is a little more than others.

### Results by using *k*NN

In order to show the importance of feature selection, we have also performed the following series experiments on the *k*NN learning machine to reduce the bias caused by learning machines.

1. KNN is a baseline method, all the genes without any selection and extraction are input into *k*NN for classification.

2. PCAKNN uses PCA as feature extraction methods, all the newly extracted components are input into *k*NN.

3. PLSKNN uses PLS as feature extraction methods, all the newly extracted components are input into *k*NN.

4. PPKNN uses PCA+PLS as feature extraction methods, all the newly extracted components are input into *k*NN.

5. GAPCAKNN uses PCA as feature extraction methods to extract new components from original gene set and GA as feature selection methods to select feature subset from the newly extracted components, the selected subset is input into *k*NN.

6. GAPLSKNN uses PLS as feature extraction methods to extract new components from original gene set and GA as feature selection methods to select feature subset from the newly extracted components, the selected subset is input into *k*NN.

7. GAPPKNN uses PCA+PLS as feature extraction methods to extract new components from original gene set and GA as feature selection methods to select feature subset from the newly extracted components, the selected subset is input into *k*NN.

Since there are parameters for *k*NN, we try to reduce its effect to our comparison and use three parameters for *k*NN, they are *k *= 1, *k *= 4 and *k *= 7.

It is noted that different data sets need different optimal parameters for different methods, we do not choose the optimal parameters, because we do not exhibit the top performance of one special method on one single data set, but we want to show the effect of our proposed framework.

#### Prediction performance

The average error rates and the corresponding standard deviation values are shown in Table 3, from which we can find the similar observations:

**Table 3:** Statistical classification error rates (and their corresponding standard deviation) by using *k*NN with different different parameters on four microarray data sets (%)

DATASET	KNN	PCAKNN	GAPCAKNN	PLSKNN	GAPLSKNN	PPKNN	GAPPKNN
*k *= 1 for *k*NN							

CNS	47.5(3.5)	43.8(8.7)	40.8(10.2)	44.9(9.3)	36.4(10.7)	44.9(10.3)	34.3(8.3)
COLON	32.5(1.4)	28.4(8.4)	27.1(10.3)	24.8(14.3)	21.9(7.5)	30.2(12.2)	18.2(7.2)
LEUKEMIA	16.1(2.2)	14.7(10.8)	11.4(8.7)	15.7(10.4)	12.3(8.6)	15.9(11.9)	8.4(6.4)
LUNG	17.6(2.3)	11.8(7.1)	11.0(4.6)	11.8(5.3)	6.1(4.7)	13.2(5.6)	7.8(4.1)
Average	28.4(2.3)	24.6(8.7)	22.57(8.4)	24.3(9.8)	19.2(7.8)	25.3(10.0)	17.1(6.5)

*k *= 4 for *k*NN							

CNS	48.6(1.2)	46.5(11.2)	41.5(11.0)	44.9(9.9)	38.4(10.8)	47.8(9.9)	38.5(8.7)
COLON	44.6(2.8)	42.9(12.9)	36.2(9.2)	35.3(14.3)	28.8(8.8)	34.9(13.7)	24.5(8.4)
LEUKEMIA	32.5(1.9)	31.5(14.1)	28.1(11.9)	15.5(9.6)	14.8(14.9)	18.6(11.5)	10.0(7.8)
LUNG	16.2(0.8)	15.8(4.6)	13.5(4.8)	12.6(6.3)	10.1(4.8)	13.4(6.5)	9.4(3.5)
Average	35.4(1.7)	34.1(10.7)	28.8(9.2)	27.0(10.0)	23.0(8.0)	28.67(10.4)	20.6(7.1)

*k *= 7 for *k*NN							

CNS	46.5(1.4)	39.1(6.6)	38.6(6.8)	41.4(10.3)	39.4(9.4)	39.0(9.0)	31.9(7.6)
COLON	30.9(1.0)	28.4(8.4)	26.0(8.3)	28.3(11.6)	24.3(8.3)	25.8(11.0)	19.5(7.2)
LEUKEMIA	24.3(1.0)	22.7(10.2)	17.7(9.1)	13.5(9.0)	11.5(8.8)	12.4(8.0)	7.0(6.2)
LUNG	16.3(0.6)	13.2(3.6)	10.1(3.5)	11.5(4.1)	6.5(4.7)	12.1(5.4)	7.5(3.61)
Average	29.5(1.0)	25.8(7.2)	23.1(6.9)	23.6(8.7)	20.4(7.8)	22.42(8.3)	16.4(6.2)

• Results of all the classification methods with feature selection and extraction like PLSKNN, GAPLSKNN, PCAKNN, GAPCAKNN, GAPPKNN are better than that of KNN without any other dimension reduction on average and on each cases.

• Results of classification methods with feature selection like GAPLSKNN, GAPCAKNN and GAPPKNN are better than those of the corresponding feature extraction methods without feature selection like PLSKNN, PCAKNN and PPKNN on average and each cases.

• Different from results by using SVM, results of GAPPKNN are better than those of PCAKNN and GAPCAKNN, even the corresponding results of PLSKNN and GAPLSKNN on average. Only on the Lung data set out of four data sets, GAPLSKNN obtains the best results than other methods do.

• Compared with results by using SVM, on the CNS, Colon and Leukemia data sets, results by using *k*NN are better, while on the Lung data set, results by *k*NN are worse. But we can not compare learning machines by these results because we did not optimize the parameters.

#### Number of selected features

We also show the number of features selected by each method in Table 4, where the values for PCAKNN means the ratios of the number of top principle components to that of extracted components, those of PLSKNN and PPKNN have the same meaning. The values for GAPCAKNN means the ratios of the number of selected components used in *k*NN to that of extracted components, and those of GAPLSKNN and GAPPKNN have the same meaning.

**Table 4:** Average percentage of features (and their corresponding standard deviation) used by *k*NN with different parameters on four microarray data sets (%)

DATASET	PCAKNN	GAPCAKNN	PLSKNN	GAPLSKNN	PPKNN	GAPPKNN
*k *= 1 for *k*NN						

CNS	68.5(6.5)	32.3(8.0)	69.2(8.0)	32.2(6.4)	62.5(7.3)	32.3(9.1)
COLON	78.2(4.4)	29.7(7.8)	58.3(5.2)	32.8(6.4)	61.4(9.0)	34.2(7.2)
LEUKEMIA	68.0(8.8)	28.6(6.2)	47.8(7.1)	31.2(7.6)	54.3(8.3)	33.3(6.8)
LUNG	73.4(6.2)	72.2(7.6)	78.4(7.2)	68.9(5.9)	79.8(8.1)	71.9(6.9)
Average	72.0(6.5)	40.7(7.4)	63.4(6.9)	41.2(6.6)	64.5(8.2)	42.9(7.5)

*k *= 4 for *k*NN						

CNS	71.2(6.8)	26.6(7.9)	68.2(7.8)	31.6(9.5)	62.4(9.8)	23.1(8.2)
COLON	80.3(7.5)	32.2(6.8)	59.7(5.2)	27.3(8.3)	62.3(8.8)	32.5(7.5)
LEUKEMIA	81.4(6.9)	26.7(5.7)	46.8(8.8)	35.5(7.1)	50.7(7.3)	33.2(6.0)
LUNG	78.2(8.7)	71.2(6.3)	74.7(6.0)	69.3(4.1)	80.2(8.9)	70.0(6.2)
Average	77.7(7.5)	39.1(6.7)	62.3(6.9)	40.9(7.2)	63.9(8.7)	39.7(7.0)

*k *= 7 for *k*NN						

CNS	72.8(7.1)	29.4(8.2)	61.7(8.8)	32.3(6.1)	68.1(10.3)	32.0(8.8)
COLON	81.2(8.3)	25.7(7.7)	52.4(6.3)	36.7(5.7)	65.4(8.9)	33.4(7.3)
LEUKEMIA	79.1(6.9)	28.9(6.5)	48.9(9.1)	32.1(6.8)	51.3(9.1)	32.7(6.1)
LUNG	80.0(5.2)	72.5(8.7)	78.3(7.4)	62.9(7.6)	82.7(9.2)	80.9(5.8)
Average	78.2(6.9)	39.1(7.8)	60.3(7.9)	41.0(6.6)	66.8(9.4)	44.7(7.0)

From Table 4, we can see that if we use the top components as in PCAKNN, PLSKNN and PPKNN, about 60–80% components are selected into learning machines, while if we use feature selection to select useful components as in GAPCAKNN, GAPLSKNN and GAPPKNN, about 30% components are selected on average. Only on the LUNG data set, the selected by different methods are 70–80% of extracted components.

#### Distribution of selected features

**Figure 3:**
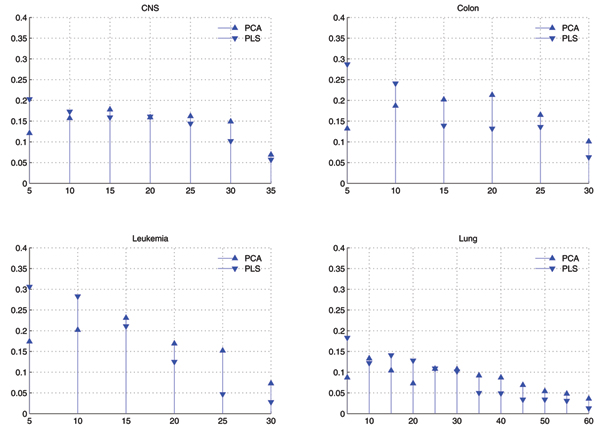
**Comparison of distributions of eigenvectors used by GAPCAKNN and GAPLSKNN with *k *= 1 for *k*NN**. X-axis corresponds to the eigenvectors in descending order by their eigenvalues and has been divided into bins of size 5. Y-axis corresponds to the average value of times that eigenvectors within some bin are selected by GA.

**Figure 4:**
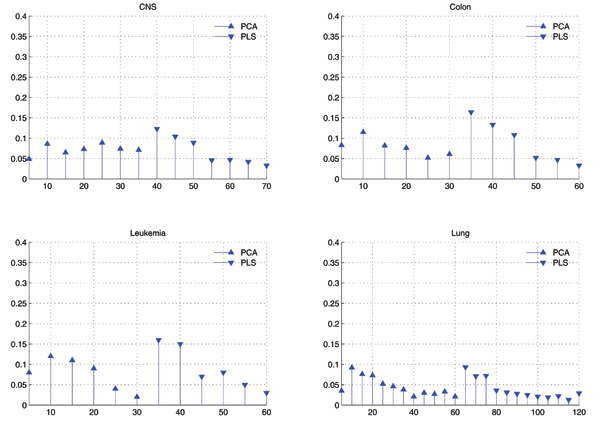
**Comparison of distributions of eigenvectors used by GAPPKNN with *k *= 1 for *k*NN**. X-axis corresponds to the eigenvectors in descending order by their eigenvalues and has been divided into bins of size 5. Y-axis corresponds to the average value of times that eigenvectors within some bin are selected by GA.

Fig. 3 shows the comparison of distributions of components selected by GA in two cases of GAPCAKNN and GAPLSKNN, and Fig. 4 shows that of GAPPKNN. Difference between Fig. 3 and Fig. 4 is that in Fig. 3, PCA and PLS are used as feature extraction individually, while in Fig. 4, PCA is combined with PLS as feature extraction methods.

From Fig. 3 and Fig. 4, we can find the similar observations as below:

• When only PLS is used for feature extraction, the top components are more than that of others in the selected components, but the others are also selected, the top, the more.

• When only PCA is used, the top components is less than others in the selected features, and the tail components are more important than others.

• When both PCA and PLS are used as feature extraction methods, they are nearly equal in the selected components, and the top components of PLS are a little more than others.

## Discussion

The results are interesting, beyond our imagination, but they are reasonable.

From the experimental results, we know not the top components are important. The reason can be found in the subsection of feature extraction. For PCA, components are extracted by maximizing the variance of a linear combination of the original genes, , but not maximizing the discriminative power for classifiers like support vector machine (SVM) and *k *nearest neighbor (*k*NN). Therefore, the top component of PCA is not the top one with high discriminative power of classifiers. For PLS, components are extracted by maximizing the covariance between the response variable ***y ***and the original genes *X*, $w_{q}=\arg\max_{w^{t}w=1}(\text{Cov}(Xw,y))$. Therefore, the top component of PLS is more important than the others for classifiers. Furthermore, the top components of PCA are not the top feature subset with high discriminative power for classifiers, while the top ones of PLS are the top feature subset with high discriminative power, but the tail ones have also discriminative power, they are selected too. So, we should not only choose the top components, but employ feature selection methods to select a feature subset from the extracted components for classifiers.

Feature selection is performed by genetic algorithm (GA), which shows great power to select feature subsets for classifiers, this can be seen from the experimental results. Here genetic algorithm based feature selection is a so called wrapper model, which uses the classifier to measure the discriminative power of feature subsets from the extracted components. This method has been proved the best one feature selection method [16]. While this wrapper method is time consuming, nowadays, the scale of data sets is increasing rapidly, so efficient feature selection methods need be developed.

Partial least squares is superior to principle component analysis as feature extraction methods. The reason is simple, PLS extracts components by maximizing the covariance between the response variable **y **and the original genes *X*, which considers using the labels **y **and can be viewed as a supervised method. While PCA extracts components by maximizing the variance of a linear combination of the original genes, which does not consider using the label **y **and can be viewed as an unsupervised method. Here, we try to improve the classification accuracy of SVM, this is a supervised task, so PLS a supervised method is superior to PCA, an unsupervised method.

Features selected by different classifiers has minor difference, and results of prediction accuracy are also different. Feature selection has done more effect on *k*NN than that on SVM. Because *k*NN is more sensitive on high dimensional data sets than SVM. But, they all benefit from feature selection.

## Conclusion

We have investigated a systematic feature reduction framework by combing feature extraction with feature selection. To evaluate the proposed framework, we used four typical data sets. In each case, we used principle component analysis (PCA) and partial least squares (PLS) for feature extraction, GA as feature selection, support vector machine (SVM) and *k *nearest neighbor (*k*NN) for classification. Our experimental results illustrate that the proposed method improves the performance on the gene expression microarray data in accuracy. Further study of our experiments indicates that not all the top components of PCA and PLS are useful for classification, the tail component also contain discriminative information. Therefore, it is necessary to combine feature selection with feature extraction and replace the traditional feature extraction step as a new preprocessing step for analyzing high dimensional problems.

## Methods

### A novel framework of dimension reduction

**Figure 5:**
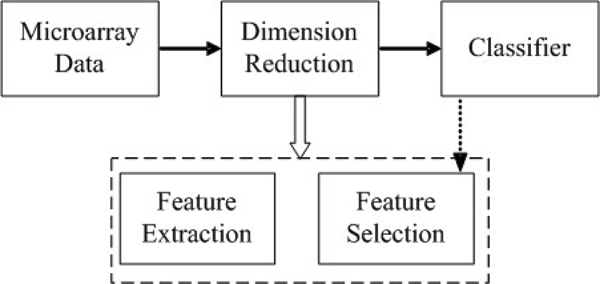
A framework of dimension reduction for the analysis of gene microarray data.

Principle Components Analysis (PCA) and Partial Least Squares (PLS) are two favorite methods in gene analysis, but how to determine the number of extracted components for classifiers is a critical problem. In the previous works, the number is fixed as 3 or 5 top ones, or obtained by cross validation. These works assume that only the top several components are important. In fact the components are ranked from a statistical view; it may not the same rank according to their discriminative ability. Therefore, we propose to apply feature selection techniques to select components for classifiers. Fig. 5 illustrates the main steps of the framework employed here. The main difference from the traditional approach is the inclusion of a step that performs feature selection among the features extracted by feature extraction methods. From Fig. 5, we can see that dimension reduction consists of two parts, feature extraction and feature selection, here feature extraction is performed by PCA and PLS, feature selection is performed by GA and classifier is performed by support vector machine (SVM) or *k *nearest neighbor (*k*NN). In Fig. 5, classifier is also applied to feature selection, that is also so call the wrapper evaluation strategy, classification performance of classifiers is used to evaluate the selected feature subset. These are explained in detail as follows.

### Feature extraction

#### Principle component analysis

PCA is a well-known method of dimension reduction [17]. The basic idea of PCA is to reduce the dimensionality of a data set, while retaining as much as possible the variation present in the original predictor variables. This is achieved by transforming the *p *original variables *X *= [***x***_**1**_, ***x***_**2**_,..., ***x***_***p***_] to a new set of *q *predictor variables, *T *= [***t***_**1**_, ***t***_**2**_,..., ***t***_***q***_], which are linear combinations of the original variables. In mathematical terms, PCA sequentially maximizes the variance of a linear combination of the original predictor variables,

(1) $$u_{q}=\underset{u^{T}u=1}{\arg\max}(\text{Var}(Xu))$$

subject to the constraint $u_{i}^{T}S_{X}u_{j}=0$, for all 1 ≤ *i *<*j*. The orthogonal constraint ensures that the linear combinations are uncorrelated, *i.e*. Cov(*X***u**_*i*_, *X***u**_*j*_) = 0, *i *≠ *j*. These linear combinations

(2) *t*_*i *_= *X***u**_*i*_

are known as the principal components (PCs). Geometrically, these linear combinations represent the selection of a new coordinate system obtained by rotating the original system. The new axes represent the directions with maximum variability and are ordered in terms of the amount of variation of the original data they account for. The first PC accounts for as much of the variability as possible, and each succeeding component accounts for as much of the remaining variability as possible. Computation of the principal components reduces to the solution of an eigenvalue-eigenvector problem. The projection vectors (or called the weighting vectors) ***u ***can be obtained by eigenvalue decomposition on the covariance matrix *S*_*X*_,

(3) *S*_*X*_**u**_*i *_= *λ*_*i*_**u**_*i*_

where *λ*_*i *_is the *i*-th eigenvalue in the descending order for *i *= 1,..., *q*, and **u**_*i *_is the corresponding eigenvector. The eigenvalue *λ*_*i *_measures the variance of the *i*-th PC and the eigenvector **u**_*i *_provides the weights (loadings) for the linear transformation (projection).

The maximum number of components *q *is determined by the number of nonzero eigenvalues, which is the rank of *S*_*X*_, and *q *≤ min(*n*, *p*). But in practical, the maximum value of *q *is not necessary. Some tail components, which have tiny eigenvalues and represent few variances of original data, are often needed to be reduced. The threshold of *q *often determined by cross-validation or the proportion of explained variances [17]. The computational cost of PCA, determined by the number of original predictor variables *p *and the number of samples *n*, is in the order of min(*np*^2 ^+ *p*^3^, *pn*^2 ^+ *n*^3^). In other words, the cost is *O*(*pn*^2^+ *n*^3^) when *p *> *n*.

#### Partial least squares based dimension reduction

Partial Least Squares (PLS) was firstly developed as an algorithm performing matrix decompositions, and then was introduced as a multivariate regression tool in the context of chemometrics [18,19]. In recent years, PLS has also been found to be an effective dimension reduction technique for tumor discrimination [11,12,20], which denoted as Partial Least Squares based Dimension Reduction (PLSDR).

The underlying assumption of PLS is that the observed data is generated by a system or process which is driven by a small number of latent (not directly observed or measured) features. Therefore, PLS aims at finding uncorrelated linear transformations (latent components) of the original predictor features which have high covariance with the response features. Based on these latent components, PLS predicts response features **y**, the task of regression, and reconstruct original matrix *X*, the task of data modeling, at the same time.

The objective of constructing components in PLS is to maximize the covariance between the response variable **y **and the original predictor variables *X*,

(4) $$w_{q}=\underset{w^{T}w=1}{\arg\max}(\text{Cov}(Xw,y))$$

subject to the constraint $w_{i}^{T}S_{X}w_{j}=0$, for all 1 ≤ *i *<*j*. The central task of PLS is to obtain the vectors of optimal weights **w**_*i *_(*i *= 1,..., *q*) to form a small number of components, while PCA is an "unsupervised" method that utilizes the *X *data only.

To derive the components, [***t***_**1**_, ***t***_**2**_,..., ***t***_***q***_], PLS decomposes *X *and **y **to produce a bilinear representation of the data [21]:

(5) $$X=t_{1}w_{1}^{T}+t_{2}w_{2}^{T}+...+t_{q}w_{q}^{T}+e$$

and

(6) $$y=t_{1}v_{1}^{T}+t_{2}v_{2}^{T}+...+t_{K}v_{q}^{T}+f$$

where ***w***'s are vectors of weights for constructing the PLS components ***t ***= *Xw*, ***v***'s are scalars, and ***e ***and ***f ***are the residuals. The idea of PLS is to estimate ***w ***and ***v ***by regression. Specifically, PLS fits a sequence of bilinear models by least squares, thus given the name partial least squares [18].

At each step *i *(*i *= 1,..., *q*), the vector ***w***_*i *_is estimated in such a way that the PLS component, ***t***_*i*_, has maximal sample covariance with the response variable **y **subject to being uncorrelated with all previously constructed components.

The first PLS component ***t***_1 _is obtained based on the covariance between *X *and **y**. Each subsequent component *t*_*i *_(*i *= 2,..., *q*), is computed using the residuals of *X *and **y **from the previous step, which account for the variations left by the previous components. As a result, the PLS components are uncorrelated and ordered.

The number of components *q *is the only parameter of PLS which can be decided by user [11,12], by cross-validation [13] or by the regression goodness-of-fit [22]. With the increase of *q*, the explained variances of *X *and **y **are expanded, and all the information of original data are preserved when *q *reaches the rank of *X*, which is the maximal value of *q*.

Like PCA, PLS reduces the complexity of microarray data analysis by constructing a small number of gene components, which can be used to replace the large number of original gene expression measures. Moreover, obtained by maximizing the covariance between the components and the response variable, the PLS components are generally more predictive of the response variable than the principal components.

PLS is computationally efficient with cost only at *O*(*npq*), *i.e*. the number of calculations required by PLS is a linear function of *n *or *p*. Thus it is much faster than the method of PCA for *q *is always less than *n*.

### Feature selection

Finding out the optimal feature subset according to classification performance is referred to as feature selection. Given a set of features, the problem is selecting a subset that leads to the least classification error. A number of feature selection methods have been studied in the bioinformatics and machine learning fields [6-8]. There are two main components in every feature subset selection system: the search strategy used to pick the feature subsets and the evaluation method used to test their goodness based on some criteria. Genetic algorithm as a search strategy is proved to be the best one among different complete and heuristic methods [16]. There are two categories of evaluation strategies: 1) filter and 2) wrapper. The distinction is made depending on whether feature subset evaluation is performed using the learning algorithm employed in the classifier design (i.e., wrapper) or not (i.e., filter). Filter approaches are computationally more efficient than wrapper approaches since they evaluate the goodness of selected features using criteria that can be tested quickly. This, however, could lead to non-optimal features, especially, when the features dependent on the classifier. As result, classifier performance might be poor. Wrapper methods on the other hand perform evaluation by training the classification error using a validation set. Although this is a slower procedure, the features selected are usually more optimal for the classifier employed. Here we want to improve classification performance, and use the wrapper strategy. Classification performance of SVM or *k*NN is used as the criteria in this paper.

#### Genetic algorithm

Genetic Algorithm (GA) is a class of optimization procedures inspired by the biological mechanisms of reproduction. [23]. GA operate iteratively on a population of structures, each one of which represents a candidate solution to the problem at hand, properly encoded as a string of symbols (e.g., binary). Three basic genetic operators guide this search: selection, crossover, and mutation. The genetic search processes it iterative: evaluating, selecting, and recombining strings in the population during each iteration until reaching some termination condition.

The basic idea is that selection probabilistically filters out solutions that perform poorly, choosing high performance solutions to concentrate on or exploit. Crossover and mutation, through string operations, generate new solutions for exploration. Given an initial population of elements, GA uses the feedback from the evaluation process to select fitter solutions, generating new solutions through recombination of parts of selected solutions, eventually converging to a population of high performance solutions.

In our proposed algorithm GA-FS (Genetic Algorithm based Feature Selection), we use a binary chromosome with the same length as the feature vector, which equals 1 if the corresponding feature is selected as the input, and 0 if the feature is discarded. The goal of using GA here is to use fewer features to achieve the same or better performance. Therefore, the fitness evaluation contains two terms: 1) Classification error; 2) The number of selected features. We use the fitness function shown below:

(7) fitness = error + γ*number_of_selected_features,

where error corresponds to the classification error on the validation data set *D*_*v*_, *γ *is a trade-off between classification error and the number of selected features. Here between classification error and feature subset size, reducing classification error is our major concern, so *γ *is set to 1 = (2 * 10^4^).

**Figure 6:**
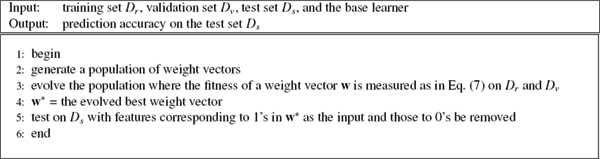
Genetic algorithm based feature selection.

The GA-FS approach is summarized in Fig. 6, where the data set is divided into 3 parts, training set *D*_*r*_, validation set *D*_*v *_and test set *D*_*s *_as in the subsection of experimental setting.

#### Classifier – support vector machines

Support vector machines (SVM) proposed by Vapnik and his co-workers in 1990s, have been developed quickly during the last decade [24], and successfully applied to biological data mining [6], drug discovery [25,26], etc. Denoting the training sample as {(*X*, **y**)} ⊆ {ℝ^*p *^× {-1, 1}}^*n*^, SVM discriminant hyperplane can be written as

***y ***= sgn(⟨**w**·*X*⟩ + *b*)

where *n *is the size of training sample, **w **is a weight vector, *b *is a bias. According to the generalization bound in statistical learning theory [27], we need to minimize the following objective function for a 2-norm soft margin version of SVM

(8) $$\begin{array}{ll} {\text{minimize}}_{w,b} & \langle w\cdot w\rangle +C\sum_{i=1}^{n}\xi_{i}^{2}\\ \text{subject to} & y_{i}(\langle w\cdot X_{i}\rangle +b)\geq 1-\xi_{i},i=1,...,n. \end{array}$$

in which, slack variable *ξ*_*i *_is introduced when the problem is infeasible. The constant *C *> 0 is a penalty parameter, a larger *C *corresponds to assigning a larger penalty to errors.

By building a Lagrangian and using the Karush-Kuhn-Tucker (KKT) complementarity conditions [28,29], we can obtain the value of optimization problem (8). Because of the KKT conditions, only those Lagrangian multipliers, *α*_*i*_s, which make the constraint active are non zeros, we denote these points corresponding to the non zero *α*_*i*_s as *support vectors *(sv). Therefore we can describe the classification hyperplane in terms of *α *and *b*:

$$y=\mathrm{sgn}\left(\sum_{i\in sv}\alpha_{i}K(X_{i},X)+b\right),$$

where *K*(**x**, **z**) is a kernel function [30], it is introduced to SVM to treat nonlinear cases, and Gaussian kernel function

*K*(**x**, **z**) = exp(-||**x **- **z||**^2^/*σ*^2^)

is considered as a prior choice [31].

#### Classifier – k nearest neighbor

*k *nearest neighbor is a non-parametric classifier [32], where the result of new instance is classified based on majority of *k *nearest neighbor category, any ties can be broken at random. It does not use any model to fit and only based on the training data set.

### Experimental data sets

Eight microarray data sets are used in our study which are listed in Table 5. They are briefly described as below.

**Table 5:** Microarray data sets used for comparison

Data Sets	Samples	Class Ratio	Features
CNS	60	21/39	7,129
Colon	62	22/40	2,000
Leukemia	72	25/47	7,129
Lung	181	31/150	12,533

**CNS **[33] developed a classification system based on DNA microarray gene expression data derived from patient samples of Embryonal tumors of the central nervous system (CNS). The data set used in our study contains 60 patient samples with 7,129 genes, 21 are survivors and 39 are failures.

**Colon **[2] used Affymetrix oligonucleotide arrays to monitor expressions of over 6,500 human genes with samples of 40 tumor and 22 normal colon tissues. Expression of the 2,000 genes with the highest minimal intensity across the 62 tissues were used in the analysis.

**Leukemia **[1] consists of 72 bone marrow samples with 47 ALL and 25 AML. The gene expression intensities are obtained from Affymetrix high-density oligonucleotide microarrays containing probes for 7,129 genes.

**Lung **[34] proposed a data set for the purpose of classifying lung Cancer between malignant pleural mesothe-lioma (MPM) and adenocarcinoma (ADCA) of the lung. The data set includes 181 tissue samples (31 MPM and 150 ADCA). Each sample is described by 12,533 genes.

### Experimental settings

To evaluate the performance of the proposed approach, we use the hold out validation procedure. Each data set is used as a whole set, originally split data sets are merged, and then we randomly divide the whole set into the training set and test set *D*_*s *_(2/3 for training and the rest for test). Furthermore, if a validation data set is needed, we splits the training data set, keeping 2/3 samples for training *D*_*r *_and the rest for validation *D*_*v*_. Classification error of SVM is obtained on the test data sets *D*_*s*_. We repeat the process 50 times.

The parameters of GA is set by default as in the software of MATLAB, and we set different parameters for SVM and *k*NN to test how parameters affect the results.

## Competing interests

The authors declare that they have no competing interests.

## Authors' contributions

Guo-Zheng Li proposed the idea and designed the algorithm, Hua-Long Bu and Mary Qu Yang performed the experiments: Jack Y. Yang conceived the project research. Guo-Zheng Li and Xue-Qiang Zeng wrote the manuscript. All co-authors contributed to the research, have read and approved the manuscript.
